# Relationship between burnout and serum markers in military personnel in plateau and plain areas of China: a cross-sectional study

**DOI:** 10.3389/fpubh.2025.1684717

**Published:** 2025-12-03

**Authors:** Lei Shi, Peng-Yan Song, Mingzhi Wang, Shen Xin, Dan-Ni Li, Qin Sun, Ke Li, Ye Tian, Yuan Wang

**Affiliations:** 1 Department of Medical Research, The Ninth Medical Center, Chinese PLA General Hospital, Beijing, China; 2 Department of Outpatient, The Ninth Medical Center, Chinese PLA General Hospital, Beijing, China; 3 Department of Emergency, The Ninth Medical Center, Chinese PLA General Hospital, Beijing, China; 4 Department of Critical Care Medicine, The Ninth Medical Center, Chinese PLA General Hospital, Beijing, China

**Keywords:** burnout, BDNF, serotonin, neuropeptide Y, military personnel

## Abstract

**Introduction:**

Burnout, as a significant factor influencing the career development of military personnel, has garnered increasing attention from military decision-makers. Military personnel stationed in plateau areas exhibit unique occupational characteristics due to prolonged exposure to specific environmental conditions. This study aimed to investigate the characteristics of burnout and serum markers among military personnel in both plateau and plain regions, thereby elucidating the relationship between burnout and serum markers while considering the impact of environmental factors.

**Methods:**

This study conducted a cross-sectional survey involving 384 military personnel (Average age 23.14 ± 5.13) from both plateau and plain regions in China between June and December 2024, utilizing random stratified cluster sampling methods. The Maslach Burnout Scale was employed to evaluate burnout among the military personnel, while serum levels of brain-derived neurotrophic factor(BDNF), neuropeptide Y(NPY), and serotonin (5-HT) were quantified using commercial ELISA kits. One-way analysis of variance and independent sample t-tests were employed to examine the differences in burnout across various variables, while regression analysis was performed to identify the factors influencing burnout.

**Results:**

The findings indicate that the overall level of burnout among military personnel is significantly elevated. Notably, the prevalence of burnout in military personnel stationed in plateau areas (100%) surpasses that observed in plain areas (96.6%). There were significant differences in the concentrations of burnout, BDNF, NPY and 5-HT among different environmental groups (*t* = −7.808 ~ 15.655, *p* < 0.01), and correlation analysis revealed that burnout was positively correlated with NPY (*r* = 0.531, *p* < 0.01), while exhibiting a negative correlation with BDNF and 5-HT (*r* = 0.537 ~ 0.608, *p* < 0.01). In addition, serum levels of NPY, and 5-HT can predict the level of burnout (*p* < 0.01). In addition, environmental factors, as stressors, can affect the level of burnout through serum serotonin and NPY.

**Discussion:**

This study combines objective serological indicators with subjective questionnaire evaluations to provide a more accurate assessment of burnout. This method can more accurately reflect an individual’s level of burnout and provide valuable experience and insights for improving the professional efficiency of military personnel in plateau environments and formulating targeted career development strategies.

## 1 Introduction

Burnout, a prevalent psychological syndrome, is characterized by emotional exhaustion, depersonalization, and diminished personal accomplishment, and this phenomenon has been extensively investigated across various occupational groups (1). As the main carrier of military struggle, soldiers shoulder the important mission of safeguarding the security of the country and the people. However, being in a high-pressure and high-risk working environment for a long time, soldiers are facing the challenge of burnout. A meta-analysis study shows that the prevalence of high burnout among military personnel ranges from 0.9 to 40% (2), and burnout is related to the working environment (3, 4).

Due to its unique geographical environment and climatic conditions, the plateau region presents significant challenges to both the physical and mental health of soldiers stationed there for extended periods. Environmental factors such as plateau hypoxia, intense ultraviolet radiation, and substantial diurnal temperature variations not only impact physiological functions but may also profoundly affect mental well-being. A study by Alcantara-Zapata indicated that exposure to plateaus can influence an individual’s stress levels (5). Factors including low oxygen availability and reduced atmospheric pressure are associated with negative emotional states, which can manifest as heightened depression and anxiety (6). Research has demonstrated that adverse emotional experiences contribute to the extent of burnout experienced by individuals (7). Prolonged exposure to these harsh environmental conditions can easily result in physical fatigue, psychological stress, and diminished job satisfaction, ultimately culminating in burnout (8).

The phenomenon of burnout is influenced by a multitude of factors, including work-related stress, social support systems, and individual psychological resilience (9). Recent studies have increasingly demonstrated that burnout transcends mere psychological implications; it may also be associated with various biological alterations, particularly changes in specific serum biomarkers (10). From a physiological perspective, when an individual encounters a stressful situation, there are significant changes in the body’s internal environment. The endocrine system responds with heightened activation of the hypothalamic–pituitary–adrenal (HPA) and hypothalamic–pituitary-thyroid (HPT) axes, which is accompanied by marked immune responses and the secretion of anabolic or catabolic hormones (11). The influence of plateau environments on the physical and mental health of soldiers is significant and warrants attention. Hypoxic conditions may lead to heightened oxidative stress and inflammation, potentially affecting serum marker levels (12, 13). However, due to variations in individuals’ psychological resilience, life experiences, and various psychosocial factors, those who endure prolonged stress may experience these physiological reactions differently (14).

Serum markers, including brain-derived neurotrophic factor (BDNF), interleukin-1 (IL-1), interleukin-6 (IL-6), serotonin (5-HT), and neuropeptide Y (NPY), have been shown to play significant roles in the regulation of stress response, and the neuroimmune system (15, 16). Scholars have found that individuals with lower serum BDNF exhibit higher levels of burnout than those with higher serum BDNF (17). Deneva et al. (18) identified a significant association between salivary cortisol and glycated hemoglobin in whole blood with burnout within the medical community, and among the teaching population, individuals experiencing higher levels of burnout exhibited reduced cortisol secretion (19). Therefore, we hypothesize that these markers may have a potential influence on burnout. Tao et al.’s research has demonstrated that the environment significantly affects serum marker (20), a conclusion that has also been corroborated by other studies (21–23).

In conclusion, the relationship between environmental factors and chronic stress-induced burnout, as well as their association with serum markers, remains ambiguous. Nevertheless, existing research has established a clear connection between environmental influences and serum markers, in addition to a distinct link between serum markers and burnout. However, current studies exploring the relationship between environmental conditions and burnout are still limited. Therefore, we propose that serum markers may play a crucial role in mediating the effects of environmental factors on burnout. To further investigate this hypothesis, we conducted research on burnout and serum markers in both plateau and plain regions to elucidate the impact of plateau environments on human serum markers and clarify the relationship between these biomarkers and burnout.

## 2 Methods

### 2.1 Study population

This study was conducted from June 2024 to December 2024. It involved a cross-sectional survey of Chinese military personnel stationed in plateau areas above 3,000 meters and plain areas around 50 meters. The primary focus was on conventional combat units such as infantry, while excluding special mission types like special forces and paratroopers. Research subjects were selected using random stratified cluster sampling methods. Specifically, participants assigned numerical identifiers to 42 designated units included in the survey through a computer-generated random number system. Subsequently, units were randomly selected from smallest to largest in proportion according to different regional classifications as the final objects of the survey (rounded to the nearest whole number). In total, military personnel from 12 units participated in this study: four out of 12 units located in Lhasa, Xizang; two out of seven units in Golmud, Qinghai; four out of 16 units in Beijing; and two out of seven units in Shijiazhuang, Hebei.

It is generally accepted that the sample size should be at least five times the number of projects under investigation. Therefore, our research required a minimum of 75 participants. A total of 393 military personnel from these 12 camps took part in interviews during which questionnaires were distributed as part of data collection efforts. Individuals not affiliated with the military were excluded from participation in this investigation. Questionnaires with missing values greater than four were discarded (those with fewer than three missing values were imputed), resulting in a total of 384 valid questionnaires being obtained, yielding an effective recovery rate of 97.7% (384/393). Among the respondents, there were 357 males and 27 females aged between 18 and 52 years old, with an average age calculated at (23.14 ± 5.13) years old. The research subjects comprised active-duty military personnel as well as civilian staff associated with the People’s Liberation Army of China.

### 2.2 Measurement methods

#### 2.2.1 General information

The general information scale is an instrument developed independently by the researchers, which includes variables such as gender, age, educational background, duration of military service, and working hours.

#### 2.2.2 Maslach burnout inventory-general survey (MBI-GS)

The Chinese version of the Maslach Burnout Inventory-General Survey (MBI-GS) was employed to evaluate burnout symptoms across various occupational groups (24). The MBI-GS was translated and revised by Chinese psychologists (25), resulting in a tool that comprises 15 items categorized into three dimensions: emotional exhaustion (EE, 5 items), depersonalization (DP, 4 items), and personal accomplishment (PA, 6 items). A 7-point Likert scale was utilized for scoring, ranging from 0 (never) to 6 (every day), reflecting the subjective feelings associated with each item.

Scores for each dimension were calculated as the average of all items within that dimension. The overall burnout score (OB) is computed using the formula OB = 0.4*EE + 0.3*DP + 0.3*PA and based on these scores, burnout severity is classified into three levels: no burnout (0–1.49), moderate burnout (1.50–3.49), and severe burnout (3.50–6.00), (26). In this study, an OB score of ≥1.49 was considered indicative of burnout. Furthermore, the Cronbach’s alpha coefficient for the MBI-GS in this survey was found to be 0.89, indicating good internal consistency.

#### 2.2.3 Sample collection and measurement of serum markers

We collected fasting blood samples from participants at military bases located on both the plateau and the plain. At 8:00 a.m., during the sample collection process, a practicing physician and a nurse stationed at the monitoring facility obtained fasting blood samples. These samples were placed in EDTA tubes and centrifuged at 5000 RPM for 5 min immediately to ensure contamination-free specimens and to mitigate any risk to personnel involved in the process. Serum samples were subsequently transferred to Eppendorf tubes and stored in a −4 °C transfer chamber for transportation to the laboratory within 8 h. The samples were then maintained strictly at −80 °C until the day of analysis.

The levels of cortisol, BDNF, neuropeptide Y, serotonin, IL-1, and IL-6 in the resulting samples were analyzed using enzyme-linked immunosorbent assay (ELISA) according to the instructions provided in the DUMABIO kit catalog based in Yunnan, China (Cortisol ELISA kit/BDNF ELISA kit/NPY ELISA kit/5-HT ELISA kit/ IL-1 ELISA kit/IL-6 ELISA kit, DUMABIO, Yunnan, China). Absorbance readings were recorded with a ChroMate microplate reader P4300 (Awareness Technology Instruments, Palm City, FL, United States). Among the various ELISA kits employed, the minimum detection concentration for all kits is less than 1 nmol/L, and the coefficient of variation both within and between plates is below 15%.

During the testing process, laboratory technicians were blinded to the source and information pertaining to the samples. Each sample was assigned a numerical identifier for testing purposes, and the test results were subsequently correlated by investigators with the assessment outcomes derived from each participant’s questionnaire. Another laboratory technician oversaw the quality control management of the testing process. Each sample was measured simultaneously in duplicate, with the difference between the two measurements remaining below 10%. The average value was recorded, and no anomalous results were reported.

#### 2.2.4 Statistical analysis

The data collation for this study was meticulously validated by the researchers to ensure the accurate entry of questionnaire responses. Statistical analysis was conducted using SPSS V25.0, primarily encompassing the following tasks:

Initially, we evaluated whether all variables adhered to a normal distribution. Descriptive analyses of categorical variables were performed using frequency and percentage, while mean and standard deviation (SD) were employed for continuous variables. Independent sample *t*-tests and one-way analysis of variance (ANOVA) were utilized to compare differences in burnout levels across various categorical variables. The independent sample *t*-test primarily identifies differences between two variables, whereas one-way analysis of variance (ANOVA) is utilized to assess differences among three or more variables. The results of pairwise comparisons following the one-way analysis of variance were adjusted using the Bonferroni correction.

Subsequently, linear regression analysis was conducted to identify potential influencing factors associated with burnout, and variance inflation factor (VIF) were used to detect collinearity between variables.

The mediation model was evaluated utilizing the PROCESS macro program (Model 4) for SPSS, developed by Hayes. Parameter estimation was conducted using the bias-corrected non-parametric percentile bootstrap method. A total of 5,000 random samples were employed to generate bootstrap confidence intervals in order to assess the significance of the indirect effects. If the 95% confidence interval (CI) does not encompass zero, both the coefficients and indirect effects are deemed significant.

All *p*-values were calculated as two-tailed, with a significance level established at 0.05.

## 3 Results

### 3.1 Descriptive

In the plains, the majority of personnel consisted primarily of active duty military members (66.7%), with a predominance of males (86.7%). Most were over 23 years old (55.0%) and unmarried (56.7%). The educational background revealed that 40% had completed high school, while 60.0% had less than 8 years of military service experience. Additionally, 58.3% reported daily working hours of fewer than 8 h. Among the participants, 6 individuals did not report any subjective burnout, whereas 174 indicated experiencing moderate levels of subjective burnout.

In the plateau area, most respondents were also active duty military personnel (75.0%), with an overwhelming majority being male (98.5%). A significant portion was under the age of 23 (69.1%) and single (83.8%). Regarding education, 58.8% held a bachelor’s degree, and a notable percentage had served for less than 8 years in the military (88.2%). The average burnout score recorded was 2.47 with a standard deviation of 0.339; all personnel reported experiencing moderate levels of burnout.

Among various demographic characteristics analyzed regarding differences in burnout levels across groups studied—the results indicated statistically significant differences solely attributed to gender and average working hours (seen in Table 1).

**Table 1:** Changes in burnout among military personnel in plateau and plain areas with different demographic characteristics.

Variables	Plain areas			Plateau areas		
	*N* (%)	Burnout/means (SD)	*t/F*	*N* (%)	Means (SD)	*t/F*
Identity						
Active-duty military personnels	120 (66.7)	2.09 (0.476)	0.704	153 (75.0)	2.44 (0.349)	−1.088
Civilian staff members	60 (33.3)	2.00 (0.409)		51 (25.0)	2.55 (0.302)	
Gender						
Male	156 (86.7)	2.10 (0.450)	2.014*	201 (98.5)	2.47 (0.339)	7.387**
Female	24 (13.3)	1.76 (0.383)		3 (1.5)	2.17 (0.167)	
Age						
≦23y	81 (45.0)	2.06 (0.482)	−0.050	141 (69.1)	2.48 (0.356)	0.567
>23y	99 (55.0)	2.06 (0.437)		63 (30.9)	2.43 (0.303)	
Marital status						
Unmarried	102 (56.7)	2.04 (0.470)	−0.320	171 (83.8)	2.47 (0.340)	0.092
Married	78 (43.3)	2.08 (0.439)		33 (16.2)	2.50 (0.349)	
Education background						
High school^(1)^	72 (40)	2.00 (0.395)	0.408	36 (17.6)	2.50 (0.389)	0.180
College or Bachelor^(2)^	54 (30)	2.13 (0.505)		120 (58.8)	2.48 (0.345)	
Master or Doctor^(3)^	54 (30)	2.07 (0.395)		48 (23.5)	2.43 (0.299)	
Length of Military Service						
≦8 years	108 (60.0)	2.03 (0.449)	−0.668	180 (88.2)	2.47 (0.342)	0.383
>8 years	72 (40.0)	2.11 (0.465)		24 (11.8)	2.43 (0.339)	
Smoking						
Yes	66 (36.7)	2.18 (0.488)	1.556	84 (41.2)	2.49 (0.366)	0.694
No	114 (63.3)	1.99 (0.424)		120 (58.8)	2.43 (0.298)	
Average working hours (per day)						
≤8 h^(1)^	105 (58.3)	1.73 (0.153)	5.856**	87 (57.4)	2.35 (0.298)	10.455**
8 ~ 9 h^(2)^	30 (16.7)	2.04 (0.475)^#^		48 (23.5)	2.49 (0.312)^#^	
≥9 h^(3)^	45 (25.0)	2.32 (0.396)^#&^		39 (19.1)	2.79 (0.287)^#&^	
Severity of burnout						
Non	6 (3.2)	1.40 (0.103)		0 (0)		
Moderate	174 (96.8)	2.08 (0.444)		204 (100)	2.47 (0.339)	
Severe	0 (0)			0 (0)		
TOTAL	180	2.06 (0.454)		204	2.47 (0.339)	

** for *P* < 0.01; * for *P* < 0.05; ^#^ for compare with (1), *P* < 0.05; ^&^ for compare with (2), *P* < 0.05; SD for standard deviation; *t* for independent sample *t*-test; *F* for one-way analysis of variance.

### 3.2 Comparison of burnout and serum markers in different environmental groups

In our comparison of burnout and serum markers across different environmental populations, we observed statistically significant differences in burnout, BDNF, serotonin (5-HT), and neuropeptide Y (NPY) between the plateau and plain military groups (*p* < 0.01). However, no significant differences were found for IL-1, IL-6, or cortisol (COR), *p* > 0.05 (seen in Table 2).

**Table 2:** Comparison of burnout and serum markers in different environmental groups.

Variables	OB	5-HT (ng/L)	NPY (ng/L)	BDNF (ng/L)	IL-1 (ng/L)	IL-6 (ng/L)	COR (μg/L)
Plain Areas	2.06 (0.454)	126.44 (10.853)	126.01 (10.907)	404.17 (11.657)	20.86 (1.755)	229.06 (21.370)	236.95 (20.173)
Plateau Areas	2.47 (0.339)	118.57 (9.949)	140.47 (10.040)	366.16 (15.290)	20.44 (1.884)	226.97 (21.400)	235.37 (23.250)
*t*	−5.884	−4.256	−7.808	15.655	1.303	0.554	0.409
*P*	<0.01	<0.01	<0.01	<0.01	0.195	0.581	0.683

OB for Overall Burnout; 5-HT for serotonin; BDNF for brain-derived neurotrophic factor; NPY for neuropeptide Y; IL for interleukin; COR for cortisol.

### 3.3 Correlation analysis

Pearson correlation analysis showed that burnout was positively correlated with neuropeptide Y (*p* < 0.01) and negatively correlated with BDNF and serotonin (*p* < 0.01), but there was no correlation between burnout and the levels of interleukin-1, interleukin-6, and cortisol. Table 3 illustrates the correlations between all continuous variables examined in this study.

**Table 3:** Correlation analysis among burnout and serum markers.

Variables	OB	5-HT	NPY	BDNF	IL-1	IL-6	COR
Overall Burnout (OB)	—						
Serotonin (5-HT)	−0.608**	—					
Neuropeptide Y (NPY)	0.531**	−0.579**	—				
brain-derived neurotrophic factor (BDNF)	−0.537**	0.555**	−0.585**	—			
Interleukin-1 (IL-1)	−0.073	0.160	−0.111	0.227	—		
Interleukin-6 (IL-6)	0.088	0.074	−0.022	0.119	0.281**	—	
Cortisol (COR)	−0.136	−0.016	0.054	−0.008	0.122	0.054	—

** for *P* < 0.01; B for unstandardized coefficients; β for standardized coefficients; VIF for variance inflation factor; *R*^2^ for explanatory power; Δ*R*^2^ for adjusted *R*^2^.

### 3.4 Multiple linear regression analysis

To account for the potential effect of demographic factors on the results, we performed multiple linear regression analyses with demographic variables (including gender and average working hours per day) and Environment as control variables (shown statistical differences as described in Section 3.1), burnout as the dependent variable, and serum markers (shown associations as described in Section 3.3) as independent variables. A VIF of >5 indicates high correlation of the variables (27), the findings indicate that the collinearity among each variable is inadequate. And the results showed that neuropeptide Y and serotonin (5-HT) could predict the level of burnout (seen in Table 4).

**Table 4:** Multiple linear regression analysis of burnout.

Variables	*B*	*β*	*t*	VIF
Gender (man for 1, female for 2)	−0.087	−0.050	−0.807	1.080
Average working hours per week (≤8 h for 1, 8 ~ 9 h for 2, ≥9 h for 3)	0.020	0.036	0.593	1.029
Environment (plain for 1, Plateau for 2)	0.341	0.383	4.145**	2.377
BDNF	0.002	0.087	0.388	2.798
Neuropeptide Y	0.009	0.248	3.541**	1.370
Serotonin (5-HT)	−0.016	−0.389	−5.396**	1.451
*F*			26.285**	
*R* ^2^			0.566	
*ΔR* ^2^			0.544	

** for *P* < 0.01.

### 3.5 Testing the mediating role of environment and burnout

We conducted a mediating effect analysis, considering environmental factors as independent variables, burnout as the dependent variable, and neuropeptide Y and serotonin as intermediary variables. The results indicated that both serotonin and neuropeptide Y mediated the relationship between environmental factors and burnout, the proportion of the mediating effects of serotonin and NPY in environmental factors and burnout were 28.15 and 15.45%, respectively (shown in Table 5).

**Table 5:** The mediating effect of environment and burnout.

Paths	Bootstrap effects					
	Effect	SE	P	LLCI	ULCI	Mediating effect
Total effect	0.5285	0.0639	0.001	0.4020	0.6550	
Direct effect						
Environmental factors → burnout	0.2981	0.0611	0.001	0.1772	0.4190	56.40%
Indirect effect						
Environmental factors → serotonin → burnout	0.1488	0.0407		0.0823	0.2439	28.15%
Environmental factors → NPY → burnout	0.0817	0.0327		0.0297	0.1601	15.45%

LLCI, lower limit 95% confidence interval; ULCI, upper limit 95% confidence interval; NPY, neuropeptide Y.

## 4 Discussion

In our study, we systematically analyzed the characteristics of burnout among individuals with varying demographic profiles. We examined the differences in burnout levels and serum markers across different environmental groups and explored the relationship between serum markers and burnout.

Our analysis of burnout within populations defined by demographic characteristics revealed that variations in working hours were closely associated with levels of burnout. Additionally, gender differences exhibited distinct patterns, which align with findings from other occupational groups, such as nurses (28) and physicians (29, 30).

This study investigates the impact of environmental factors on burnout and serum markers. Numerous studies have established the relationship between environmental changes and internal conditions of body, particularly in unique settings such as plateau regions (31, 32). However, reports addressing the influence of environmental factors on burnout remain relatively scarce. Previous research conducted in various settings has consistently demonstrated that military personnel experience significantly higher levels of burnout in specialized work environments compared to those in urban areas (33). Some researchers have examined the characteristics of burnout and serum markers in soldiers stationed in arid regions of Xinjiang, China. In comparison to their counterparts in urban areas, soldiers deployed in arid desert environments exhibited significantly elevated levels of burnout (34).

In our study, we observed a higher prevalence of burnout among military personnel stationed in the plateau region. All participants from this area reported moderate levels of burnout, while 96.6% (174 out of 180) of those in the plains region also indicated experiencing burnout. In our preliminary research, we also observed a similarly high incidence rate (35). This phenomenon may be attributed to the occupational characteristics inherent to military service, which often involve extended working hours, substantial workloads, and significant responsibilities—factors that are known contributors to burnout (20). Furthermore, the organizational culture within military settings that prioritizes discipline and obedience may lead to feelings of isolation among personnel and a perceived lack of autonomy, these conditions can diminish job satisfaction, impair combat effectiveness, and elevate turnover rates as potential stressors affecting military members’ well-being (36).

From the perspective of burnout levels, military personnel stationed in plateau regions exhibit a higher degree of burnout compared to their counterparts in plain regions. This disparity can be attributed to the unique environmental conditions characteristic of plateau areas. The harsh plateau environment, marked by low oxygen levels, extreme temperatures, and intense ultraviolet radiation, significantly impacts both the physical and mental well-being of military personnel (37). Research indicates that factors such as reduced oxygen availability and lower atmospheric pressure are associated with negative emotional states, including heightened depression and anxiety (38). Furthermore, these adverse emotional experiences can exacerbate the extent of burnout individuals encounter (7). Prolonged exposure to such challenging environments is likely to result in physical fatigue, psychological stress, and diminished job satisfaction—ultimately culminating in increased burnout rates among soldiers (8).

On the other hand, our study revealed that serum markers can also be influenced by environmental factors. The levels of neuropeptide Y were found to be elevated in military personnel stationed in plateau regions, whereas the level of brain-derived neurotrophic factor (BDNF) and serotonin was observed to be lower. This variation may correlate with individual differences in coping mechanisms related to burnout. Specifically, higher levels of burnout are associated with intensified feelings of stress and an amplified physiological response to stressors. Serotonin, neuropeptide Y, and BDNF have been identified as key serum markers indicative of the body’s response to stress (8, 39, 40). These findings further substantiate the notion that environmental conditions can serve as stimuli that induce stress responses in individuals.

Notably, this study proposes that serum serotonin functions as a crucial neurotransmitter involved in the stress response, playing an essential role in the regulation of neural activity. By modulating the hypothalamic–pituitary–adrenal (HPA) axis, it significantly influences an individual’s response to stress (10). Environmental stressors are perceived and transmitted through sensory pathways to various structures within the central nervous system (CNS), including the thalamus and limbic regions such as the amygdala and hippocampus, along with cortical areas primarily located in the prefrontal cortex (PFC). Direct projections from the thalamus to the amygdala may provide an initial representation of stimuli, while norepinephrine stimulation from the locus coeruleus (LC) enhances this stimulus, thus initiating a significant stress response (41), however, serotonin may reduce the severity of this reaction. Neuropeptide Y has emerged as a prominent research focus; evidence indicates that it rapidly activates neurons with neuropeptide Y receptors in response to stress, thereby alleviating anxiety—a process associated with reduced activity within the HPA axis (42). This discrepancy may be attributed to the stress resilience factors inherent in military personnel (43). Morgan demonstrated that uncontrollable stress can lead to a significant increase in plasma NPY levels in humans, particularly among certain special forces units (44). Consequently, as manifestations of chronic stress, environmental stimuli can influence levels of burnout through alterations in serotonin and neuropeptide Y levels.

In our research, we found that the prevalence of burnout among military personnel is notably high, particularly for those stationed in plateau regions. Consequently, it is recommended that military managers implement a comprehensive approach combining psychological assessments and physiological indicators during annual physical examinations for screening purposes. For military personnel identified with significant abnormalities through this screening process, timely intervention measures should be enacted. This can be accomplished by arranging rotational rest periods to temporarily remove them from high-pressure work environments, thereby allowing them to obtain essential rest and adjustment time. Simultaneously, a robust psychological counseling and intervention framework should be established. Professional psychologists ought to provide individualized one-on-one counseling and support aimed at alleviating stress and restoring psychological equilibrium. Such measures will better equip military personnel to fulfill their duties while maintaining optimal mental and physical health.

## 5 Limitations and strengths

In summarizing the results of this study, we acknowledge several limitations. First, as a cross-sectional study, it can only provide insights into potential causal explanations. Therefore, future research should consider longitudinal approaches to further elucidate the causal relationships between the variables. Secondly, there may be sampling bias in this study, particularly given that female military personnel may be underrepresented in our sample. Although the male-to-female ratio aligns with the occupational characteristics and realities of military personnel, gender as a potential influencing factor could introduce errors into the results. Similarly, factors such as sleep patterns and the duration of acclimatization to plateaus may exert comparable influences. Thirdly, while we endeavored to minimize subjective selection of participants by employing a comprehensive sampling method, biases inherent to the population may still have been introduced. Additionally, due to constraints related to manpower and resources, we selected some units from both plateau and plain regions for our survey; thus limiting the generalizability of our findings across all military personnel in various environmental contexts within China. Furthermore, our investigation did not account for potential factors affecting burnout—such as physiological changes during adaptation to plateau environments—which suggests that addressing these factors will necessitate more comprehensive follow-up studies in the future.

However, it is important to note that our study utilized rigorously validated instruments specifically designed to meet most of our research needs and demonstrated robust psychometric properties in assessing study outcomes. Moreover, this research provides valuable insights into the relationship between burnout and serum markers among military personnel. It also contributes significantly to existing literature by clarifying distinctions between burnout and serum markers across different settings within military populations.

## 6 Conclusion

This study revealed significant differences in burnout levels and serum markers among military personnel based on gender and daily working hours across various environmental settings. Notably, the level of burnout experienced by military personnel stationed in plateau regions was found to be higher than that of their counterparts in plain areas. Furthermore, we observed distinct variations in serum BDNF, serotonin, and neuropeptide Y levels among military personnel from different environmental contexts. Through our analysis, we established a negative correlation between military burnout and serum BDNF levels, while finding a positive correlation with both serum serotonin and neuropeptide Y levels. Moreover, environmental factors, as stressors, can affect the degree of military burnout through the concentrations of serum serotonin and neuropeptide Y. The results of this study provide valuable empirical insights for enhancing the professional effectiveness of military personnel in plateau environments and formulating targeted career development strategies. Furthermore, this study provides a serological framework for more accurately predicting the job burnout status of military personnel.

## Data Availability

The raw data supporting the conclusions of this article will be made available by the authors, without undue reservation.
